# Association of asthma and herpes zoster, the role of vaccination: A literature review

**DOI:** 10.1002/iid3.718

**Published:** 2022-10-11

**Authors:** Abhigan Babu Shrestha, Tungki Pratama Umar, Yasmine Adel Mohammed, Manjil Aryal, Sajina Shrestha, Unnat Hamal Sapkota, Lukash Adhikari, Shumneva Shrestha

**Affiliations:** ^1^ M Abdur Rahim Medical College Dinajpur Bangladesh; ^2^ Faculty of Medicine Sriwijaya University Palembang Indonesia; ^3^ Faculty of Medicine Assiut University Assiut Egypt; ^4^ KIST Medical College Patan Nepal; ^5^ Patan Academy of Health Science Lalitpur Nepal; ^6^ Maharajgunj Medical Campus, Institute of Medicine Tribhuvan University Kathmandu Nepal

**Keywords:** asthma, chickenpox, herpes zoster, Shingrix, vaccine, varicella zoster virus

## Abstract

Herpes Zoster (HZ) is the reactivation of a previous infection with varicella‐zoster virus (VZV) which shares the same mode of transmission as HZ. It presents with painful erythematous vesicles in a dermatome which is characterized by a burning sensation before and after the rash. Any conditions with suppressed cellular immunity example diabetes mellitus, chronic obstructive pulmonary disease, asthma, cardiovascular diseases, chronic steroid uses, malignancy, etc. causes reactivation of the virus. Impaired immune responses in asthma patients either in any age group may increase their susceptibility to HZ infection owing to skewed Th1/Th2 immunity, resulting in predominant Th2 conditions and an unwarranted Th2 cell response against respiratory allergens. Similarly, many studies have delineated the association of asthma with HZ. However, the relation between steroid use in asthma and HZ is uncertain, its immunosuppressive effect might be responsible for increased susceptibility to the infection. As HZ increases the economic burden and morbidity, its prevention should use vaccines. There are two types of Food and Drug Administration (FDA)‐approved vaccine available against HSV one of which is given as a single dose vaccine called Zostavax, for people 50–59 years but its efficacy falls after 3rd dose and on the subsequent 4th dose and is also contraindicated in human immunodeficiency virus/acquired immunodeficiency syndrome, pregnancy and people taking immunosuppressive drugs. Shingrix is preferred by FDA which is a two doses vaccine that is given 6 months apart for people above 50 years and to immunocompromised people. Hence, proper counseling and education about the risks of herpes should be informed to the patients with timely utilization of the vaccine.

## 1 INTRODUCTION

Varicella zoster virus (VZV) is an enveloped, double‐stranded, and linear human alpha herpes virus that is highly contagious and transmitted either by direct contact with infected lesions fluid from the vesicles or inhalational through infected respiratory droplets.^1^, ^2^ it causes chickenpox as a primary infection, and it is presented mainly by fever, tiredness, loss of appetite and characteristic itchy rash. Chickenpox is a self‐limited disease if it is adequately treated and the patient has a good immunity. However, the virus is not permanently eradicated and becomes dormant in human peripheral neuronal ganglion for several decades.^3^

Herpes zoster (HZ) is considered to be reactivated by a previous infection with VZV and nearly has the same transmission routes either by direct contact or by infected respiratory secretion. In a review by Thomas et al.,^4^ stated that the lifetime prevalence of HZ was 10%–30% and may rise to 50% in those over 85 years old; another cross‐sectional study performed in England stated that the lifetime prevalence among English population was 12.6% for female and 10.3% for males^5^ and the risk increased among those of white ethnicity and ex‐smokers.^5^

HZ is mainly presented by erythematous skin rash, which is very painful; this maculopapular rash is confined to one area of the skin called the dermatome and is usually present on one side of the body.^6^ Some people reported a burning sensation in the dermatome before the appearance of the rash; they describe it as an electric sensation.

Despite the availability of highly effective antiviral drugs, HZ is associated with many complications, including postherpetic neuralgia (PHN), Meningitis Retention Syndrome, Acute Colonic Pseudo‐obstruction (Ogilvie syndrome), Keloids and Other Types of Isotopic Response, Pseudohernia formation and cysts, Erythema Multiforme, Vasculitis, Recurrent HZ, Occult Neoplasia, HZ Opthalmicus, disseminated HZ, Stroke, and other neurological complications, VZV reactivation and even COVID‐19.^7^, ^8^

Although the exact cause of HZ reactivation is still unknown, many risk factors are thought to play a role in this reactivation; some of which are related to stress and family history, others are related to any condition causing immunosuppression or decrease in cellular immunity, such as malignancy, steroid, and aging which was explicitly found to double the risk of the disease after the age of 50^4^, ^9^, ^10^ and in another recent systematic review, the pooled analysis showed a higher risk of HZ in older age in comparison to young age (risk ratio [RR] = 1.65, 95% confidence interval [CI] = 1.37–1.97).^11^ In addition, several chronic debilitating diseases like diabetes mellitus, cardiovascular diseases, bronchial asthma, and chronic obstructive pulmonary disease were also found to be potential risk factors for HZ.^5^, ^12^

However, there have not been many detailed studies to entail an association between herpes and asthma. To our knowledge, this is the first and to date evidence of literature review providing insights regarding herpes and its association with asthma.

## 2 METHODS

The research design method of this literature review is an evidence based rapid review. The aim of this review is first to scrutinize and assess all the available articles on HZ and asthma. Standard databases (PubMed, Embase, and Google Scholar) and literature searches (Google search engine) with relevant titles: “Shingles,” “herpes zoster,” “herpes zoster vaccine,” and “asthma” from inception were implemented. Relevant articles were gathered in Google spreadsheet and extracted information was then performed by five authors (T.P.U., Y.A.M, M.A., U.H.S., A.B.S.).

## 3 ASTHMA

### 3.1 Prevalence

The global prevalence of asthma has been estimated to be between 1% and 18%.^13^ The estimated prevalence is highest in Africa (11.3%; 95% CI = 9.0%–14.1%) and lowest in South‐East Asia (8.8%; 95% CI = 7.0%–11.0%).^14^ In terms of age, children aged 5–9 years are the most commonly affected (prevalence: 13.41%), and the majority of those affected are boys from lower‐middle‐income countries.^14^ However, in adult populations, females are more likely to contract asthma, peaking at around 70 years of age.^15^

### 3.2 Pathophysiology

Despite the lack of a complete understanding of the pathophysiologic mechanisms underlying asthma progression, it is widely accepted that disruptions to the innate and adaptive immune systems play a significant role.^16^, ^17^ One of the most important mechanisms is the abnormally excessive immune response of T helper type 2 (Th2) cells, which results in uncontrolled Th2‐cell‐associated cytokines. However, according to a recent report, this change can be attributed to the impairment of other innate immune cells such as basophils and mast cells.^18^ Furthermore, the problem of the innate immune systems, such as insufficient interferon‐λ induction, have been described.^19^, ^20^ These innate immune deficiencies have been linked to a disruption in the antiviral signaling pathways.^21^, ^22^ Based on these observations, several studies have reported an increased susceptibility to viral infections, including in nonrespiratory sites, in asthmatic patients.^19^, ^23^

## 4 ASTHMA AND HZ

Association between asthma and skin infection, specifically HZ, has been proposed in several studies, both in the adult^24^, ^25^, ^26^, ^27^, ^28^, ^29^ and pediatric^30^, ^31^, ^32^ populations. In the adult populations, the prevalence of HZ sufferers who had a history of bronchial asthma ranges from 7.1% to 23%^24^, ^26^ while, in the pediatric population is around 7.7% to 28.1%. This varies according to the diagnostic guideline (lowest with Asthma Predictive Index [API] and highest with predetermined Asthma criteria [PAC]).^31^

According to a Korean study, adult patients with HZ have 1.32 times the odds of having asthma than the control group (95% CI: 1.28–1.35).^27^ A similar finding was found in Taiwan (adjusted hazard ratio/aHR: 1.48; 95% CI: 1.36–1.62) (after adjustment for sex, age, comorbidities, systemic and inhaled corticosteroids use, and annual outpatient visits),^25^ United States (odds ratio [OR]: 1.70; 95% CI: 1.20–2.42),^24^ England (OR: 1.21; 95% CI: 1.17–1.25),^26^ Germany (OR: 1.22; 95% CI: 1.18–1.26; incidence rates: 7.14),^29^ and Spain (adjusted incidence ratio/aIRR, men: 1.34; 95% CI: 1.27–1.42, women: 1.32; 95% CI: 1.28–1.37)^28^ respectively. The risk appears to be similar across age groups, with an OR of 1.24 in the <50‐year age group and 1.18 in the population aged ≥70 years.^26^ The lower ratio in Korea than in a similar study in its continent (Asia) could be attributed to a strictly matched control group regarding socioeconomic status and previous medical history.^27^

In the pediatric population in the United States, as illustrated by Wi et al.,^31^ the adjusted OR (aOR) of contracting HZ while having a history of asthma was slightly higher than in adults, ranging from 1.81 (95% CI: 1.15–2.88) when diagnosed using the PAC to 2.56 (95% CI: 1.08–6.56) when diagnosed using the original API criteria. The value increases when the API group is subjected to an adjusted analysis based on atopic status (OR: 2.85; 95% CI: 1.04–9.07). The results were similar to another study conducted in the United States (aOR: 2.09; 95% CI: 1.24–3.52).^30^ However, it is lower in a Taiwanese cohort study (HR: 1.15; 95% CI: 1.06–1.26). That study controlled for other similar variables such as age, gender, environment, and family and found that the incidence of HZ was more significant in the asthmatic cohort than in the non‐asthmatic cohort (8.85 vs. 7.05 per 10,000 person‐years). Uncontrolled asthma patients with recurrent visits or hospitalization were also found to have an increased risk of HZ incidence than well‐controlled asthma patients.^32^

### 4.1 Role of steroids

There are conflicting findings regarding the use of steroids in asthma patients and their association with HZ development. Several studies have found that when compared to those who did not have a regular controller, asthma patients who received daily inhaled corticosteroid (ICS) or systematic corticosteroid treatment had a slightly higher risk of HZ.^25^, ^26^, ^29^, ^32^ The same results were obtained when a noncorticosteroid immunosuppressive agent was used.^26^ However, combining ICS and Montelukast (leukotriene receptor antagonist)^32^ can minimize the risk. Meanwhile, studies from the United States,^24^, ^30^, ^31^ United Kingdom,^33^ and Taiwan (limited to ICS)^25^ found no association between steroid use as a controller and HZ incidence.

Impaired immune responses in asthma patients including innate and adaptive forms of cellular immunity may increase their susceptibility to HZ infection,^34^ resulting from predominant Th2 conditions and an unwarranted Th2 cell response against respiratory allergens.^35^ Conversely, asthmatics may experience reciprocal Th1 immune insufficiency. Meanwhile, Th1‐type cellular immunity is responsible for preventing varicella‐zoster virus (VZV) reactivation (as HZ) and alleviating PHN.^26^ As a result, Th1 immune deficiency in asthma patients may make them more susceptible to HZ manifestation.^36^

Impaired innate immunity in asthma patients could also increase the risk of HZ emergence. Asthma patients have impaired innate immune responses as well as mucosal defense systems, resulting in an altered lower respiratory microbiome.^37^ Likewise, the VZV is also associated with deficiencies in the antiviral properties of the innate immune system (e.g., Langerhans cells and plasmacytoid dendritic cells) due to its resistant evasion property.^38^, ^39^

Although the impact of steroid use in asthma patients on the risk of developing HZ is uncertain, its immunosuppressive effect may be responsible for increased susceptibility to the infectious process. The mechanism is primarily attributed to systemic corticosteroids’ role in inhibiting inflammatory cytokines, reducing antigen‐presenting cells, and decreasing T‐cell counts.^40^ However, as demonstrated in the majority of studies, asthma‐related medications such as corticosteroids, even at the highest doses, did not show a significant association with the development of HZ, even though statistical power to address these associations may be limited.^24^

## 5 VACCINATION

Until further studies, prevention of herpes is warranted to protect from the unwanted economic burden and increased morbidity. Currently, two types of vaccine are available on the market; Zostavax, a live‐attenuated vaccine, and another recombinant subunit vaccine called Shingrix, which contains the viral glycoprotein that helps VZV replication and cell transmission.^41^, ^42^ Both vaccines play on improving cellular immunity, but Shingrix has VZV specific antigen glycoprotein E and an adjuvant component (AS01b) that increases VZV‐specific cellular mediated immunity and enhances humoral immune response.^43^

### 5.1 Types of vaccines

Zostavax is a single‐dose vaccine approved, in 2006, by The Food and Drug Administration (FDA) to be used for the population aged 50–59 years.^44^, ^45^ A randomized clinical trial (RCT) of 22,439 subjects of the same age group (50–59 years) found the efficacy of Zostavax in reducing HZ reactivation to be 69.8% (95% CI: 54.1–80.6).^46^ However, it is no longer recommended because vaccine effectiveness after 3rd year is only slightly above 50% and is further diminished to ≤24% after the 4th year. Furthermore, it is also contraindicated in immunocompromised patients (human immunodeficiency virus/acquired immunodeficiency syndrome, malignancies), immunosuppressive drugs, and pregnancy.^47^, ^48^ Shingrix is a recombinant subunit vaccine that was approved by FDA in 2017 for adults ≥50 years and in adults aged 18 years and older with immunodeficiency or immunosuppression caused by known disease or due to therapy; it is taken in two doses 2–6 months apart.^45^ As, asthma predominately occurs in younger age and obstructive pulmonary diseases are associated with immunosuppression, it would be feasible for consideration of vaccine. But still, clinical studies must be done to confirm this hypothesis as multiple factors may be associated. In a recent RCT of 15,411 adults aged ≥50 years, the efficacy of Shingrix to prevent HZ was 96.9% (95% CI: 90.6–99.4) in people aged 50–59 years, 94.1% (95% CI: 85.6–98.1) in people aged 60–69 years, 98.3% (95% CI: 89.9–100.0) in people aged ≥70 years and the overall effect of the whole population was 96.2% (95% CI: 92.7–98.3).^49^ The adverse effects of vaccination are milder including redness, swelling at the site of injection, and other systemic events in the form of gastrointestinal symptoms, myalgia, fever and shivering.^49^

Both types of vaccines are effective and safe to use; however, the centers for disease control and prevention prefers Shingrix over Zostavax because of its stronger protection. The Advisory Committee on Immunization Practice recommends Shingrix for populations aged ≥50 years, regardless of history of Varicella infection or Zostavax vaccination, and recommends Zostavax immunocompetent population aged ≥60 years.^45^

### 5.2 Economic aspect

HZ imposes a tremendous economic burden worldwide, and in a systematic review by Panatto et al, the cost of disease diagnosis and initial treatment range from $600 for uncomplicated cases to $3700 for complicated conditions such as those with PHN, in the manner outpatient and consultations cost range from nearly $800 to $1150 in uncomplicated cases and $1140 to $3560 in cases complicated with PHN.^50^ In another study by Meyers et al.,^51^ the total medical cost in uncomplicated cases was $1425 and approximately $7300 in those complicated with PHN. Its incidence has increased the total healthcare costs across the board, putting financial strain on patients, and a burden on the entire global healthcare system.

Without a doubt, the application of the universal varicella vaccination has led to a significant reduction in the incidence of varicella infection. In the United States, the incidence of Varicella infection reduced by 90% after introducing the varicella vaccine into the universal childhood vaccination schedule between 1995 and 2008.^52^ And in Germany, the incidence declined by 63% in the 0–4‐year‐old age group and by 55% in all age groups between 2005 and 2009.^53^ In a recent study performed in Norway, the annual incidence of varicella reduced to 48–59 per 100,000 population compared to 1359 per 100,000 population before the introduction of the vaccine and all vaccination strategies were cost‐effective.^54^

As per the research, Silverberg et al.^55^ found that the average onset of asthma is 9.4 years after varicella infection. In contrast, those who got the varicella vaccine had onset about 3 years following vaccination, implying a unique inflammatory immune response suppression mechanism. This finding has raised an important argument regarding the cost‐effectiveness of a universal varicella immunization program considering the economic burden of asthma over the vaccination program. The study by Ditkowsky et al.^56^ regarding this demonstrated that the vaccine program was comparatively much more cost‐effective, with no apparent delay in the onset of asthma. When the vaccination and no‐vaccination arms were compared under the assumption of delayed asthma onset, vaccination was found to be less expensive, despite increased savings from asthma without vaccination.^56^

## 6 CONCLUSION

To conclude, asthma history and HZ incidence are significantly related. The association is consistent across age and gender subgroups. As a result, adequate asthma control (as evidenced by the number of emergency department visits and hospitalizations) is critical for lowering HZ incidence and its long‐term complications, such as PHN.^27^ This step can be accomplished by improving medication adherence, educating patients and parents about the routine and continued use of medications, and guiding the proper use of inhalers.^57^, ^58^, ^59^ To decrease the risk of corticosteroid‐induced herpes reactivation during asthma treatment in elderly people, utilization of other groups of drugs is highly advisable. However, to date, proper larger studies have not been established to make a significant finding. Furthermore, because the risk of HZ infection was higher in young adults and the elderly with asthma, HZ vaccination (Shringrix) may be advantageous in a broad range of age groups.^27^ Vaccination should be made an indication for the elderly asthmatic age group as they are more susceptible. However, its role in children still plays debatable inviting randomized trials for better evidence.

## AUTHOR CONTRIBUTIONS

**Abhigan Babu Shrestha**: conceptualization. **Abhigan Babu Shrestha, Tungki Pratama Umar, Yasmine Adel Mohammed, Manjil Aryal, Unnat Hamal Sapkota, and Lukash Adhikari**: manuscript writing. **Shumneva Shrestha and Sajina Shrestha**: manuscript editing.

## CONFLICTS OF INTEREST

The authors declare no conflicts of interest.

## Data Availability

Data sharing not applicable to this article as no datasets were generated or analysed during the current study.
